# Genetic profile of African swine fever virus responsible for the 2019 outbreak in northern Malawi

**DOI:** 10.1186/s12917-020-02536-8

**Published:** 2020-08-28

**Authors:** J. N. Hakizimana, G. Kamwendo, J. L. C. Chulu, O. Kamana, H. J. Nauwynck, G. Misinzo

**Affiliations:** 1 SACIDS Africa Centre of Excellence for Infectious Diseases, SACIDS Foundation for One Health, Sokoine University of Agriculture, Morogoro, Tanzania; 2 Department of Veterinary Microbiology, Parasitology and Biotechnology, College of Veterinary Medicine and Biomedical Sciences, Sokoine University of Agriculture, Morogoro, Tanzania; 3 Department of Animal Health and Livestock Development, Ministry of Agriculture, Irrigation and Water Development, Lilongwe, Malawi; 4 Department of Food Science and Technology, College of Agriculture, Animal Sciences and Veterinary Medicine, University of Rwanda, Busogo, Rwanda; 5 Department of Applied Research and Development and Foresight Incubation, National Industrial Research and Development Agency, Kigali, Rwanda; 6 Laboratory of Virology, Faculty of Veterinary Medicine, Ghent University, Merelbeke, Belgium

**Keywords:** African swine fever virus, *Asfarviridae*, Domestic pigs, Molecular characterization, Malawi

## Abstract

**Background:**

African swine fever (ASF) is an infectious transboundary animal disease which causes high mortality, approaching 100% in domestic pigs and it is currently considered as the most serious constraint to domestic pig industry and food security globally. Despite regular ASF outbreaks within Malawi, few studies have genetically characterized the causative ASF virus (ASFV). This study aimed at genetic characterization of ASFV responsible for the 2019 outbreak in northern Malawi. The disease confirmation was done by polymerase chain reaction (PCR) followed by molecular characterization of the causative ASFV by partial genome sequencing and phylogenetic reconstruction of the *B646L* (p72) gene, nucleotide alignment of the intergenic region (IGR) between *I73R* and *I329L* genes and translation of the central variable region (CVR) coded by *B602L* gene.

**Results:**

All thirteen samples collected during this study in Karonga district in September 2019 were ASFV-positive and after partial genome sequencing and phylogenetic reconstruction of the *B646L* (p72) gene, the viruses clustered into ASFV p72 genotype II. The viruses characterized in this study lacked a GAATATATAG fragment between the *I173R* and the *I329L* genes and were classified as IGR I variants. Furthermore, the tetrameric amino acid repeats within the CVR of the *B602L* gene of the 2019 Malawian ASFV reported in this study had the signature BNDBNDBNAA, 100% similar to ASFV responsible for the 2013 and 2017 ASF outbreaks in Zambia and Tanzania, respectively.

**Conclusions:**

The results of this study confirm an ASF outbreak in Karonga district in northern Malawi in September 2019. The virus was closely related to other p72 genotype II ASFV that caused outbreaks in neighboring eastern and southern African countries, emphasizing the possible regional transboundary transmission of this ASFV genotype. These findings call for a concerted regional and international effort to control the spread of ASF in order to improve nutritional and food security.

## Background

African swine fever (ASF) is a highly contagious, deadly hemorrhagic viral disease of domestic pigs and wild boars of all breeds and ages caused by ASF virus (ASFV), a double-stranded DNA arbovirus and the only member of the family *Asfarviridae*, genus *Asfivirus* [1–3]. The virus is transmitted through a sylvatic cycle involving warthogs (*Phacochoerus africanus*) which do not develop clinical disease and soft ticks of the *Ornithodoros moubata* complex inhabiting warthog burrows [4]. This ancient sylvatic cycle specific to eastern and southern Africa occasionally leads to virus spill to domestic pigs through the tick-pig cycle that involves infected soft ticks dropped by warthogs at pig shelters [5]. After the virus is introduced to the domestic pig population, transmission between domestic pig population occurs through the transmission of the virus among domestic pigs or by feeding contaminated pig products to domestic pigs, accounting for the majority of ASF outbreaks globally [6, 7]. Recently, an additional epidemiological cycle characterized by both direct transmission between infected and susceptible Eurasian wild boar (*Sus scrofa*) and indirect transmission through carcasses in the habitat has been described and named the wild boar-habitat cycle [8]. The current geographical distribution of ASF extends across more than 50 countries in Africa, Europe and Asia and among them, 33 are countries in Africa, south of Sahara [9, 10]. The recent spread of ASF within Europe and Asia has substantially increased the global concern regarding the disease and it is considered as the most devastating disease to global domestic pig industry and food security [2].

Depending on the isolate, the ASF viral genome varies in length from about 170 and 193 kilobase pairs (kbp) and encodes between 150 and 167 open reading frames with a conserved central region and variable termini [2]. Although most of the length variations were described to be associated with the gain or loss of copies within multigene families (MGF), smaller length variations are also associated with the number of tandem repeat sequences (TRS) located at loci both within coding and intergenic regions [11]. Sequence analysis of distinct genomic regions of ASFV has proved to be very useful in identifying the origin and transmission pathways of ASF during outbreaks [12]. Based on the ASFV p72 major capsid protein gene (*B646L*), 24 distinct ASFV genotypes (I-XXIV) have been described [13, 14] and analysis of additional genes has shown to provide higher resolution to distinguish between closely related isolates. The central variable region (CVR) within the *B602L* gene has shown to provide more information about relationship between isolates at genotype, country and regional levels [15–18]. Recent studies have demonstrated the value of the tetrameric repeat sequences (TRS) located in the intergenic region between the *I73R* and *I329L* genes in determining the origin and mapping the spread of closely related ASFV isolates [19, 20]. By combining p72, *B602L* (CVR) and TRS, a high level resolution is achieved for viral discrimination despite the existence of many other markers.

In Africa South of Sahara, the existence of all the 24 ASFV p72 genotypes described to date has been demonstrated [21, 22]. Briefly, in West Africa, where there is no evidence of the existence of the ASFV sylvatic cycle, only genotype I has been reported. In Central, Eastern and Southern Africa where three ASFV transmission cycles exist, rich ASFV genotypic variability exists with all 24 (I to XXIV) ASFV genotypes [9, 13, 22–25]. In Malawi, ASF is endemic and several outbreaks have been reported to the World Organization for Animal Health (OIE) in almost all its provinces [9, 10, 22, 26]. For instance, from January 2005 to December 2018, 227 ASF outbreaks which led to 87,063 pig deaths were reported to OIE [10]. Each year, ASF is reported in different parts of the country posing a serious constraint to the development of the domestic pig industry in Malawi. Despite the regular ASF outbreaks in domestic pigs within Malawi, molecular characterization of the causative viruses has been limited, thus the ASF outbreaks patterns and ASFV genotypes mapping in the country are incomplete. Most of ASFV molecular characterization studies from Malawi were carried out more than a decade ago and previously characterized ASFV strains grouped into p72 genotypes V, VIII and XII and all domestic and sylvatic ASFV transmission cycles have been described in the country [16, 18, 26, 27]. The tick-domestic pig and the sylvatic cycles of ASFV transmission involving warthogs and ticks collected from domestic pig shelters and warthogs’ burrows have been demonstrated in Malawi [28–30]. Warthogs and bush pigs which are natural reservoirs for ASFV are commonly found in National Parks and Wildlife Reserves of Malawi [28, 29, 31, 32] and may possibly play a role in the epidemiology of ASF in the country. Proper ASF outbreak investigation and continuous molecular characterization of the responsible viral strains provide insight into the transmission dynamics of the virus, differentiation of closely related strains and identification of potential transmission routes during and after outbreaks in order to guide appropriate interventions for an effective control of ASF [33]. This study aimed at confirming and conducting molecular characterization of the ASFV responsible for the 2019 outbreak in Karonga district located in northern Malawi.

## Results

### Laboratory confirmation of ASF

All collected tissue samples belonging to 13 different domestic pigs from Karonga district included in the present study were positive for ASFV after conducting diagnostic PCR using ASFV-specific primers.

### Phylogenetic reconstruction of ASFV targeting *B646L* (*p72*) gene, TRS and CVR

**Fig. 1:**
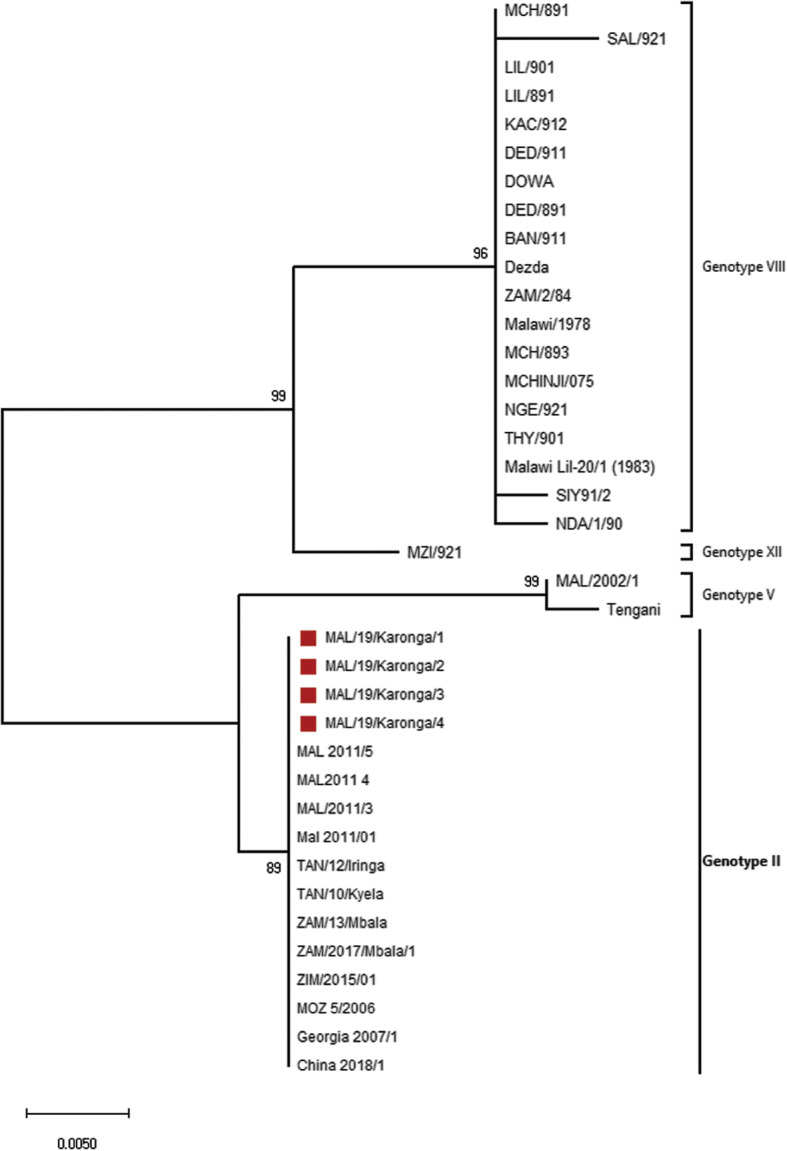
Maximum Likelihood phylogenetic reconstruction of African swine fever virus (ASFV) strains from Malawi together with genotype II ASFV previously described in Tanzania, Zambia, Mozambique, Zimbabwe, Georgia and China, sharing high nucleotide identity with viruses that caused outbreak in Malawi during September 2019 based on *B646L* (p72) gene nucleotide sequences. Phylogeny was inferred following 1000 bootstrap replications, and the node values show percentage bootstrap support. Scale bar indicates nucleotide substitutions per site. The square indicates the African swine fever virus strains that caused outbreak in Malawi during September 2019

In order to classify viruses characterized in this study among the 24 ASFV p72 known genotypes, the c-terminal end of *B646L* (p72) gene was amplified and sequenced. All sequences obtained in this study have been deposited to the GenBank and given accession numbers (Accession numbers MN755863-MN755874). The ASFV from domestic pigs in Karonga district named MAL/19/Karonga/1–4 had 100% nucleotide identity. The BLASTn of *B646L* (p72) nucleotide sequences of MAL/19/Karonga/1–4 against other ASFV strains available at GenBank showed 100% nucleotide identity with ASFV strains previously described in Tanzania, Zambia, Georgia, China, Vietnam, Estonia, Moldova, Czech Republic, Belgium and Poland. After phylogenetic reconstruction using ASFV strains indicated in Table 1, the MAL/19/Karonga/1–4 clustered together with ASFV belonging to genotype II (Fig. 1).

**Table 1:** African swine fever virus (ASFV) strains circulating in Malawi together with genotype II ASFV previously described in Tanzania, Zambia, Mozambique,Zimbabwe, Georgia and China, sharing high nucleotide identity with strains that caused outbreak in Malawi during September 2019

Isolate	Host species	Year of isolation	Town/district	Country	Accession number	P72 genotype	Reference
MAL 2011/5	Domestic pig	2011	NK^1^	Malawi	KC835275	II	Unpublished
MAL2011 4	Domestic pig	2011	NK	Malawi	JX524217	II	Unpublished
MAL/2011/3	Domestic pig	2011	NK	Malawi	KC662378	II	Unpublished
Mal 2011/01	Domestic pig	2011	NK	Malawi	JX294724	II	Unpublished
MAL/19/Karonga_1	Domestic pig	2019	Karonga district	Malawi	MN755863	II	This study
MAL/19/Karonga_2	Domestic pig	2019	Karonga district	Malawi	MN755864	II	This study
MAL/19/Karonga_3	Domestic pig	2019	Karonga district	Malawi	MN755865	II	This study
MAL/19/Karonga_4	Domestic pig	2019	Karonga district	Malawi	MN755866	II	This study
TAN/12/Iringa	Domestic pig	2012	Iringa	Tanzania	KF834193	II	[34]
TAN/10/Kyela	Domestic pig	2010	Kyela	Tanzania	JX391987	II	[35]
ZAM/13/Mbala	Domestic pig	2013	Mbala	Zambia	LC174750	II	[12]
ZAM/2017/Mbala/1	Domestic pig	2017	Mbala	Zambia	LC322016	II	[36]
ZIM/2015/01	Domestic pig	2015	Mashonaland	Zimbabwe	KX090923	II	[37]
MOZ_5/2006	Soft tick	2006	Gorongosa National Park	Mozambique	KY353984	II	[14]
Georgia 2007/1	Domestic pig	2007	Caucasus Region	Georgia	NC_044959	II	[38]
China 2018/1	Domestic pig	2018	Shenbei	China	MH722357	II	[20]
Tengani	Warthog	1960	Tengani	Malawi	AF301541	V	[16]
MAL/2002/1	Domestic pig	2002	Mpemba Camp	Malawi	AY494553	V	[27]
Malawi/1978	Domestic pig	1978	NK	Malawi	AF270707	VIII	[16]
ZAM/2/84	Domestic pig	1984	NK	Malawi	AF449471	VIII	[16]
Dezda	Domestic pig	1986	Chilikum-Wera, Dedza	Malawi	AF449479	VIII	[16]
NDA/1/90	Domestic pig	1990	Nadula	Malawi	AF449473	VIII	[16]
BAN/911	Domestic pig	1991	Bangula, Lower Shire	Malawi	AY351501	VIII	[27]
DED/891	Domestic pig	1989	Dedza District	Malawi	AY351502	VIII	[27]
DED/911	Domestic pig	1991	Mtenden Campus, Dedza	Malawi	AY351503	VIII	[27]
DOWA	Domestic pig	1986	Moya, Dowa	Malawi	AY351509	VIII	[27]
KAC/912	Domestic pig	1991	Kachendere Seminary	Malawi	AY351504	VIII	[27]
LIL/891	Domestic pig	1989	Lilongwe District	Malawi	AY351505	VIII	[27]
LIL/901	Domestic pig	1990	Kafere diptank, Lilongwe	Malawi	AY351510	VIII	[27]
MCH/891	Domestic pig	1989	Kachebere Seminary	Malawi	AY351506	VIII	[27]
MCH/893	Domestic pig	1989	Lilongwe District	Malawi	AY351507	VIII	[27]
MCHINJI/075	Domestic pig	1987	Mchinji	Malawi	AY351508	VIII	[27]
NGE/921	Domestic pig	1992	Karonga District	Malawi	AY351544	VIII	[27]
SAL/921	Domestic pig	1992	Salima District	Malawi	AY351546	VIII	[27]
SIY91/2	Domestic pig	1991	Sinyala diptank, Lilongwe	Malawi	AY351566	VIII	[27]
THY/901	Domestic pig	1990	Comforzi farm, Thyolo District	Malawi	AY351545	VIII	[27]
Malawi Lil-20/1 (1983)	Tick (pig)	1983	Chalaswa	Malawi	AY261361	VIII	[30]
MZI/921	Domestic pig	1992	Euthini, Mzinda District,	Malawi	AY351543	XII	[27]

^1^Not known

**Fig. 2:**
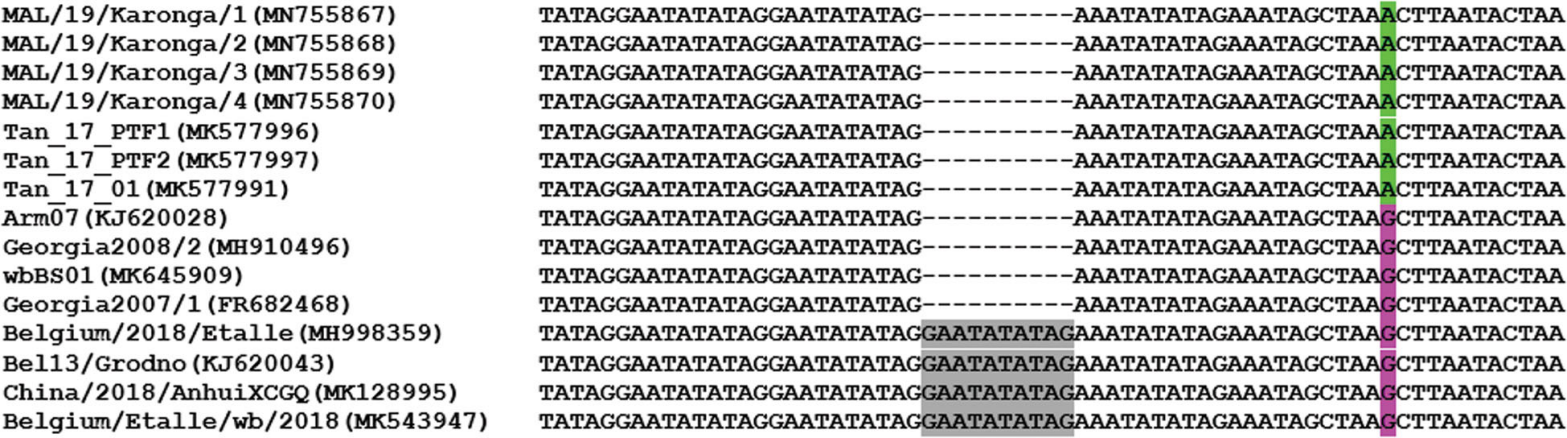
Nucleotide sequence alignment of the intergenic region between *I73R* and *I329L* genes of African swine fever virus strains belonging to *B646L* (P72) genotype II from Tanzania, Europe and China. The nucleotides highlighted in gray are absent in some viruses including the strains that caused outbreak in Malawi during September 2019. Also, a substitution of G by A is observed in Tanzanian and Malawian viruses only

The analysis of the intergenic region (IGR) between *I73R* and *I329L* genes of the strains that caused ASF outbreak in Karonga district in northern Malawi in 2019 showed 99.41% nucleotide identity with ASFV genotype II strains responsible for the 2017 outbreaks in Morogoro and Pwani regions of Tanzania and 99.16% nucleotide identity with some isolates circulating in Europe and Asia, including the Georgia 2007/1 isolate. The viruses characterized in this study lacked a GAATATATAG fragment between the *I173R* and the *I329L* genes (Fig. 2) and were classified as IGR I variants as previously described [19, 20]. In addition, a similar G to A replacement were observed in ASFV described in this study and those previously described in Tanzania (Fig. 2).

The CVR sequences obtained in this study were translated into amino acids and coded to obtain corresponding signature. The CVR tetrameric repeats of ASFV that caused the outbreak in Karonga district in 2019 included **CADT, NVDT, CASM, CAST** and **CSTS,** corresponding to B, N, D and A codes, respectively. The ASFV characterized in this study showed 10 amino acid tetramers (**BNDBNDBNAA)** that were 100% identical to each other. A similarity search against other ASFV amino acids sequences performed by BLASTp showed 100% amino acids identity to ASFV that caused previous ASF outbreaks in Tanzania, Madagascar, Zambia, Mozambique, Mauritius, Russia and China (Table 2).

**Table 2:** Selected African swine fever viruses belonging to p72 genotype II with high amino acids sequences identity with viruses collected in Malawi in September 2019 at the Central variable region (CVR) of the *B604L* gene

Virus name	Year	Country of origin	Host species	CVR Accession number	CVR signature	reference
MAL/19/Karonga_1	2019	Malawi	Domestic pig	MN755871	BNDBNDBNAA	This study
MAL/19/Karonga_2	2019	Malawi	Domestic pig	MN755872	BNDBNDBNAA	This study
MAL/19/Karonga_3	2019	Malawi	Domestic pig	MN755873	BNDBNDBNAA	This study
MAL/19/Karonga_4	2019	Malawi	Domestic pig	MN755874	BNDBNDBNAA	This study
ASFV_Tan_17_PTF2	2017	Tanzania	Domestic pig	MK276893	BNDBNDBNAA	[39]
ASFV_Tan_17_PTF1	2017	Tanzania	Domestic pig	MK276892	BNDBNDBNAA	[39]
ASFV_Tan_17_01	2017	Tanzania	Domestic pig	MK276887	BNDBNDBNAA	[39]
ASFV_Tan_15_4	2015	Tanzania	Domestic pig	MK276894	BNDBNDBNAA	[39]
ZAM/13/Mbala	2013	Zambia	Domestic pig	BAW94569	BNDBNDBNAA	[12]
ZAM/2017/Mbala/1	2017	Zambia	Domestic pig	LC322013	BNDBNDBNAA	[36]
Antani03	NK^1^	Madagascar	Domestic pig	EU649696	NK	Unpublished
Arm07	2007	Armenia	Domestic pig	JX857522	NK	[19]
MOZ_2/2006	2006	Mozambique	Tick	ATD84005	BNDBNDBNAA	[14]
MOZ/1/2002	2002	Mozambique	Domestic pig	QBG64414	NK	Unpublished
CN201801	2018	China	Domestic pig	AYD60223	NK	Unpublished
ASFV-wbBS01	2018	China	Wild boar	QAU54736	NK	Unpublished
MAD/1998	1998	Madagascar	Domestic pig	AAQ18412	NK	[16]
Tver0511/Torjo	2011	Russia	Domestic pig	AII03124	NK	[19]
Irkutsk2017	2017	Russia	Domestic pig	AUC64211	NK	Unpublished
MAU/1/2008	2008	Mauritius	Domestic pig	QBG64413	NK	Unpublished

Key: (CAST, CVST, CTST, CASI = A), (CADT, CADI, CTDT, CAGT, CVDT = B), (NVDT, NVGT, NVDI=N) and (CASM = D)

^1^Not known

## Discussion

In this study, we confirmed an ASF outbreak in Karonga district in northern Malawi that occurred during September 2019. Laboratory confirmation was done by PCR and subsequent genetic characterization of partial ASFV genome by phylogenetic reconstruction of the *B646L* (p72) gene, nucleotide alignment of the intergenic region (IGR) between *I73R* and *I329L* genes and amino acid alignment of the *B602L* (CVR) gene. After phylogenetic analysis, the ASFV strains obtained in this study clustered together with viruses belonging to ASFV p72 genotype II. Furthermore, the IGR and CVR signatures of ASFV strains obtained in this study showed high identity with p72 genotype II viruses previously described in Tanzania, Zambia, Mozambique, Zimbabwe, Georgia, China, Vietnam, Estonia, Moldova, Czech Republic, Belgium, Poland and Russia.

The nucleotide sequences of the c-terminal end of *B646L* (p72) gene of the viruses characterized in this study showed 100% nucleotide identity with those previously described in neighboring countries of Tanzania and Zambia. There is a high possibility of transboundary spread of ASFV between Malawi, Tanzania and Zambia since these countries share a common border and the towns of Karonga in Malawi, Kyela in Tanzania and Mbala in Zambia are less than 400 km apart. The ASFV strain that caused an outbreak in Kyela, Tanzania in 2010 had 100% nucleotide identity for the p72 (*B646L*) gene to Malawian ASFV collected from the 2011 outbreak in Karonga (Table 1). It was previously speculated that the introduction of ASFV genotype II, previously not described in Tanzania, occurred by importation of pig products from Karonga in Malawi [35, 36]. Since then, there has been persistent circulation of highly virulent genotype II viruses in the southern highlands of Tanzania, that have devastated nutritional and food security [40]. Since the introduction of genotype II viruses in Tanzania, these viruses have spread northwards within Tanzania causing devastating impact to the domestic pig industry and expanding the geographical range of this ASFV genotype [34, 39, 40]. Illegal transportation of infected pigs and pig products to uninfected areas have been cited to contribute to the spread of ASFV within Tanzania [34]. There is a need for higher control in order to prevent this ASFV genotype II from its northward spread otherwise it may reach other neighboring East African Community countries, such as Rwanda, Burundi, Uganda and Kenya. It is not uncommon for animal viruses to expand their geographical range as observed with peste des petits ruminants [41–43], Tilapia lake virus disease [44] and foot-and-mouth disease [45–47].

The analysis of the IGR between *I73R* and *I329L* genes showed high nucleotide identity with previously characterized ASFV genotype II strains and lacked a GAATATATAG fragment similar to ASFV strains circulating in Tanzania, different countries of Europe and Asia including the isolate Georgia 2007/1 collected in Georgia in 2007 that subsequently spread to other countries of eastern Europe and China [39, 48, 49]. In addition, we observed a similar G to A replacement in strains characterized in this study to ASFV strains responsible for the 2017 outbreaks in Morogoro and Pwani regions of Tanzania [39]. This suggest that ASFV genotype II strains circulating in Tanzania and Malawi are from probably the same source. Whole genome sequencing of strains described in this study and ASFV circulating in eastern and southern Africa will be able to discriminate closely related strains and establish more accurately epidemiological links between different ASF outbreaks occurring in the region.

The tetrameric amino acids repeats within the CVR of the *B602L* gene of the 2019 Malawian ASFV reported in this study had the signature BNDBNDBNAA which was 100% similar to the ASFV strains that caused the ASF outbreaks in Tanzania in 2017 [39] and during 2013 in Zambia [12]. The ZAM/13/Mbala virus was collected in April 2013 from domestic pigs reared in a village along the border with neighboring Tanzania and a suspected introduction from Tanzania through trans-border trade of pigs and pork products is speculated [12]. Kyela at the Tanzanian side, Karonga in northern Malawi and Mbala in Zambia share borders and ASF outbreaks have been reported to temporally coincide [12, 35, 36].

Comparison of the 3 ASFV genomic regions analyzed in this study revealed high identity between the strains characterized in this study and other viruses belonging to ASFV p72 genotype II that have caused previous outbreaks elsewhere including in Madagascar in 1998 [50]. The first ASFV reported in Madagascar was identical to the virus recovered from the 1994 ASF outbreak in Mozambique and was suspected to be the most likely source of ASF infection in Madagascar that was previously free from ASF [50, 51]. The ASFV p72 genotype II was recovered from domestic pigs in the year 2002 and from soft ticks in 2006 in Mozambique [14]. In 2007, the ASFV p72 genotype II strain with high identity to viruses previously described in Madagascar and Mozambique was reported in Mauritius for the first time and swill feeding to domestic pigs was suspected to be responsible for the introduction of the ASFV to the island [52]. The ASFV genotype II occurred in the Caucasus region of Georgia in 2007 with subsequent spread to Russia, different countries of Europe before it reached China in August 2018 and spread to neighboring Asian countries [19, 20, 49]. The virus responsible for the ASF outbreak in Georgia in 2007 was closely related to ASFV strains previously described in Mozambique, Zambia and Madagascar, thus the southern Africa countries or Madagascar were suspected to be the most likely source of ASF infection of the Georgia 2007 ASF outbreak [49]. Eastern and southern Africa countries including Malawi are characterized by the presence of wildlife protected areas with warthogs and ticks of the *Ornithodoros moubata* complex inhabiting warthogs’ burrows. These natural reservoir of the ASFV play an important role in the maintenance and transmission of the ASFV through the sylvatic cycle of the virus [14, 22, 53]. The 2015 ASF outbreak in Zimbabwe was caused by the ASFV genotype II after several years without ASF outbreak reported in the country and the transboundary spread from neighboring Mozambique was suspected [37]. The high identity between ASFV strains described in this study and viruses previously characterized in southern Africa countries, Madagascar, Europe and Asia suggests that they may have probably the same wild source and maintained through domestic cycle. In Malawi, the sylvatic cycle of ASFV involving ticks collected from warthogs habitat has been previously described [29] and a detailed study need to be carried out to assess the current role of wild suids and *Ornithodoros* ticks in the maintenance and transmission of ASFV in Malawi.

## Conclusions

The virus responsible for the 2019 ASF outbreak in Karonga district clustered into p72 genotype II and showed high nucleotide identity with ASFV strains causing outbreaks in neighboring eastern and southern Africa countries suggesting that the same ASFV strains are causing outbreaks across borders. In addition, ASFV strains described in this study were closely related to viruses previously reported in Europe and Asia. These findings highlight the need for a concerted regional and international effort to control the spread of ASF in order to improve nutritional and food security. Investigation of the role of ASFV sylvatic cycle and further characterizations by whole genome sequencing are needed to fully understand molecular epidemiology of ASFV in Malawi.

## Methods

### Study area and sample collection

**Fig. 3:**
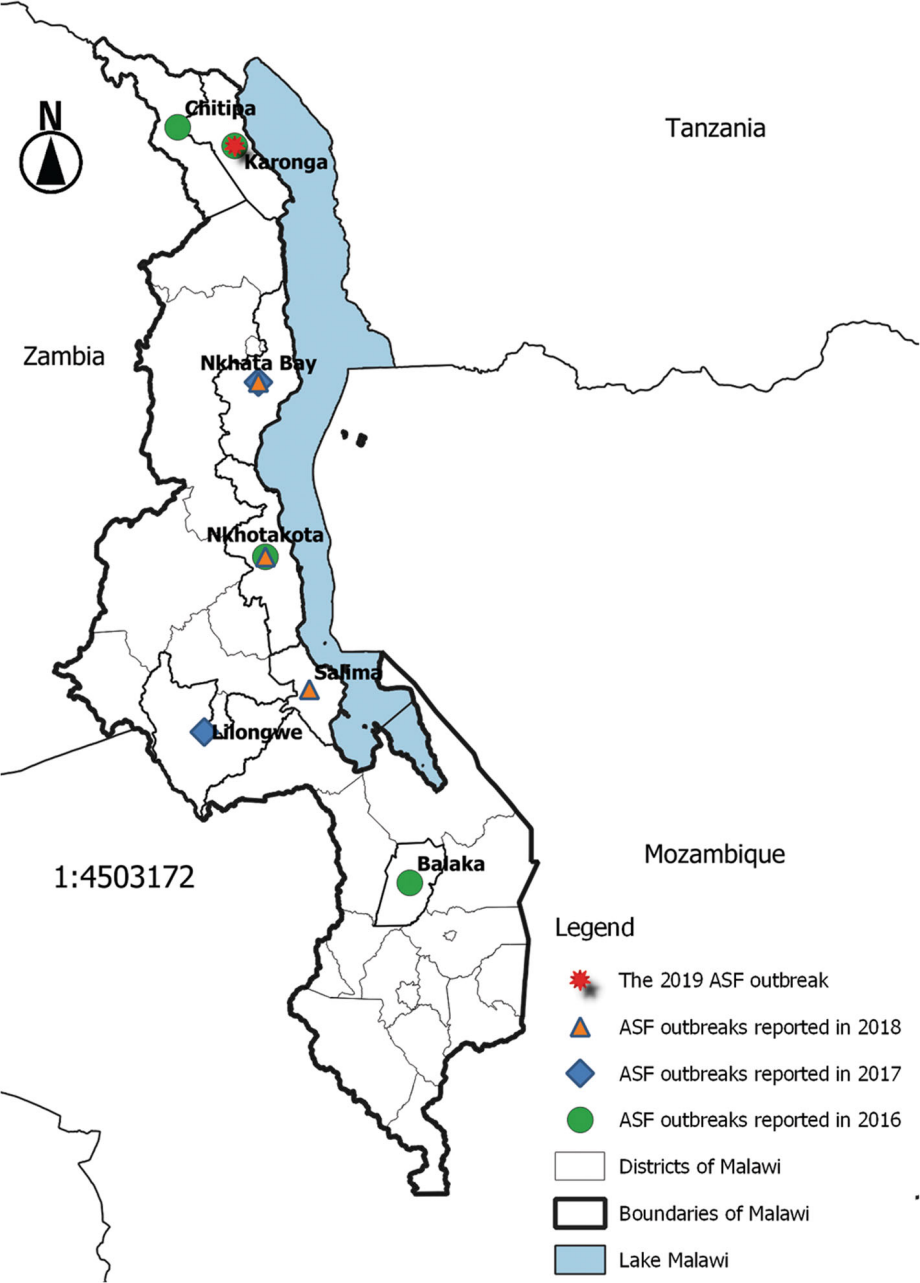
Distribution of African swine fever outbreaks reported in Malawi from 2016 to September 2019. Outbreaks occurred in all provinces of the country. Source of data: OIE World Animal Health Information System. The map was developed using QGIS version 3.4.4 (https://www.qgis.org/en/site/about/index.html)

Samples used in this study were collected in Karonga district in northern Malawi from small-scale pig farmers in response to a report from local veterinarians to the Malawian National Veterinary Epidemiology Unit of a hemorrhagic disease affecting many domestic pigs with clinical symptoms suggestive of ASF in September 2019 (Fig. 3). Two tissue samples per domestic pig including spleen and liver were aseptically collected from thirteen dead pigs and transported to the laboratory. In the laboratory, samples were processed by homogenization in sterile phosphate-buffered saline (PBS) at a ratio of 1:10 w/v followed by centrifugation at 6000 *g* for 5 min and cryopreservation of the supernatant at − 80 ^∘^C until DNA extraction.

### DNA extraction and nucleotide amplification

QIAmp nucleic acid extraction kit (Qiagen, Hilden, Germany) was used for DNA extraction from collected samples, following manufacturer’s instructions. The presence of ASFV in collected samples was confirmed by polymerase chain reaction (PCR) using ASF diagnostic primers PPA1 and PPA2, as previously described by Aguero et al. [54]. The variable 3′-end of *B646L* gene encoding the major capsid protein p72, the tetramer amino acid repeats within the hypervariable central variable region (CVR) and the intergenic region (IGR) between *I73R* and *I329L* were amplified using the following primers: p72-D/p72-U [16], ORF9L-F/ORF9L-R [55] and ECO1A/ ECO1B [19], respectively.

### Nucleotide sequencing and phylogenetic analysis

The nucleotide sequences of PCR products were obtained by automated dideoxynucleotide cycle sequencing using BigDye Terminator Cycle sequencing kit version 3.1 (Applied Biosystems, Foster City, CA). Sequence scanner software version 2.0 (Applied Biosystems, Foster City, CA) and Bioedit version 7.2.5 (Ibis Biosciences, Carlsbad, CA) were used to check the quality of raw sequences data and to obtain consensus nucleotide sequences from both forward and reverse primers for each of the amplified regions. The obtained consensus nucleotide sequences were used for BLASTn to search for similarity of nucleotide sequences obtained in this study to other nucleotide sequences available at GenBank. The dataset for p72 phylogenetic reconstruction consisted of 38 nucleotide sequences (402 characters), comprising of 4 sequences generated in this study and 34 homologous sequences from GenBank, including ASFV strains previously described in Malawi and genotype II ASFV strains from Tanzania, Zambia, Mozambique, Zimbabwe, Georgia and China, sharing high nucleotide identity with strains described in this study (Table 1). The phylogenetic tree construction was performed using Maximum Likelihood method and Kimura 2-parameter model with a bootstrap frequency of 1000 replicates as implemented by MEGA X [56]. The tandem repeat sequences (TRS) in the intergenic region between *I73R* and *I329L* genes of the strains characterized in this study were compared with other ASFV strains using CrustalW as implemented in MEGA X [56]. The central variable region nucleotide sequences of our ASFV isolates were translated and coded to obtain signatures based on previously reported codes [18, 34, 57]. A similarity search against other ASFV amino acid sequences was performed using BLASTp.

## Data Availability

The datasets generated and/or analysed during the current study are available at the GenBank repository (https//ncbi.nlm.nih.gov/genbank) with accession numbers MN755863 to MN755874.

## Abbreviations

ASF: African swine fever
ASFV: African swine fever virus
BLASTn: Basic Local Alignment Search Tool for Nucleotides
BLASTp: Basic Local Alignment Search Tool for Proteins
CVR: Central variable region
DNA: Deoxyribonucleic acid
IGR: Intergenic region
MEGA: Molecular Evolutionary Genetics Analysis
OIE: World Organization for Animal Health
P72: African swine fever major capsid protein
PBS: Phosphate-buffered saline
PCR: Polymerase chain reaction
TRS: Tandem repeat sequences
